# Supplementary material for: Critical COVID-19, Victivallaceae abundance, and celiac disease: A mediation Mendelian randomization study
Source: PLoS One. 2024 May 3;19(5):e0301998. doi: 10.1371/journal.pone.0301998 (PMC11068179; doi:10.1371/journal.pone.0301998)
Supplement: S1 File — (DOCX) [file pone.0301998.s001.docx]

Supporting information

**Critical COVID-19, *Victivallaceae* Abundance, and Celiac disease: A Mediation Mendelian Randomization Study**

S1 Fig. Scatter plots showing the effect of liability to COVID-19 on celiac disease.

S2 Fig. Leave-one-out plots of the causal effect of COVID-19 on celiac disease.

S3 Fig. Forest plot of COVID‐19 associated with celiac disease.

**S1 Fig. Scatter plots showing the effect of liability to COVID-19 on celiac disease.**

The x-axis represents the genetic association with COVID-19; the y-axis represents the genetic association with risk of celiac disease. The regression line for inverse variance weighted, weighted median, MR Egger, weighted mode and simple mode. The slope of each line corresponds to the estimated MR effect per method.

﻿Abbreviation: COVID‐19, coronavirus disease 2019; MR, Mendelian randomization;


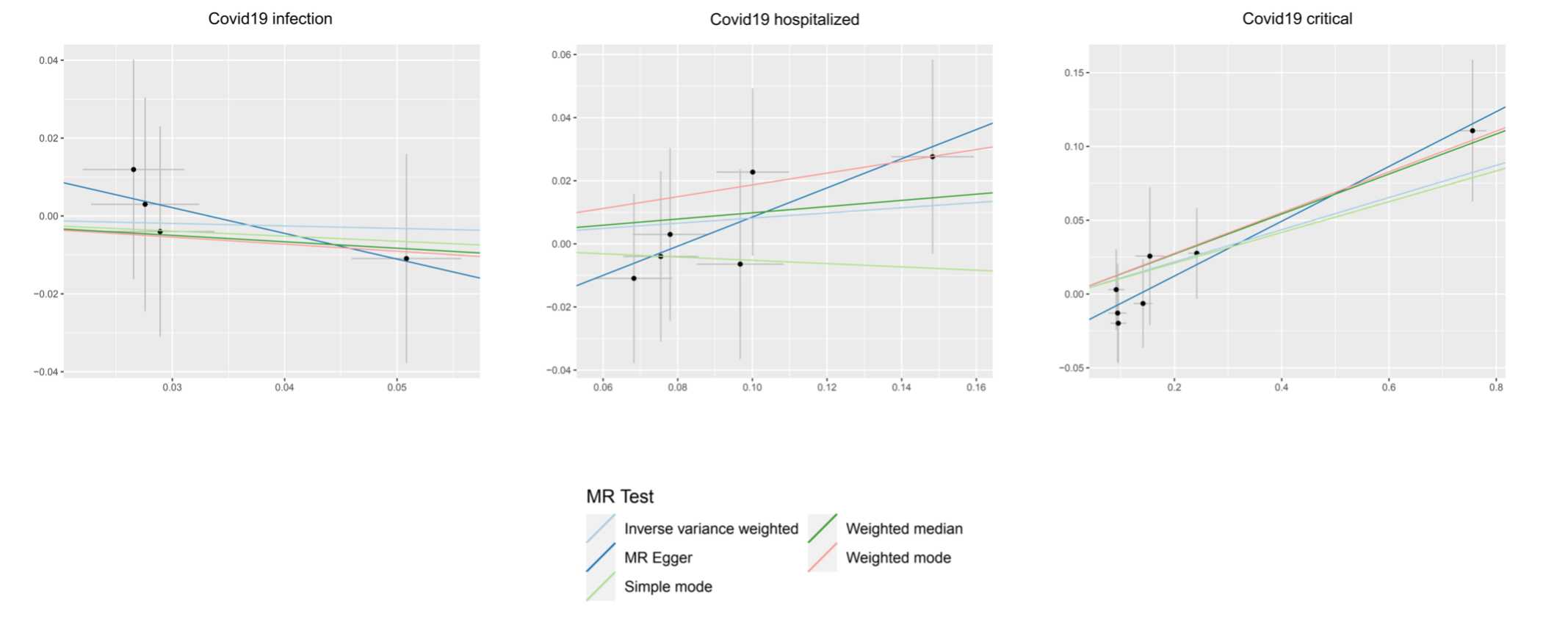


**S2 Fig. Leave-one-out plots of the causal effect of COVID-19 on celiac disease.**


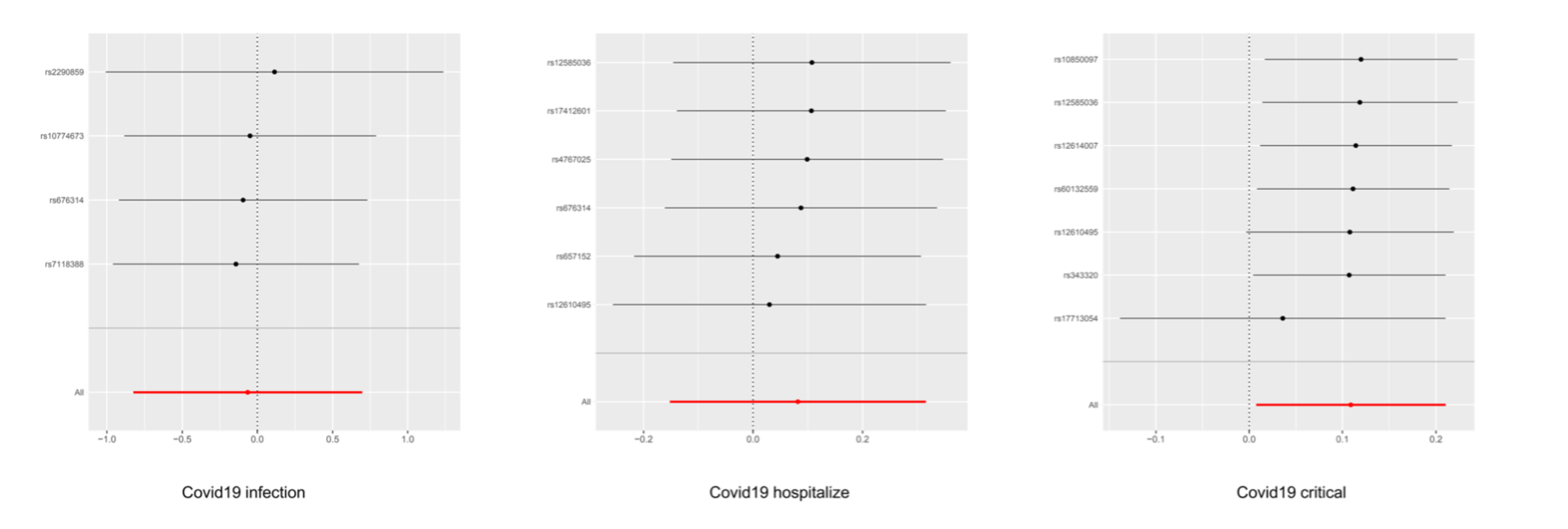


﻿The x‐axis shows leave‐one‐out sensitivity analysis for COVID‐19 on celiac disease. The y‐axis shows SNPs on COVID-19. Each *black line* represents the IVW applied to estimate the causal effect, excluding the particular variants. The *Red lines* represent the IVW estimations using all SNPs

Abbreviation: COVID‐19, coronavirus disease 2019; MR, Mendelian randomization; SNPs, single‐nucleotide polymorphisms; IVW, inverse variance weighted;

**S3 Fig. ﻿Forest plot of COVID‐19 associated with celiac disease.**


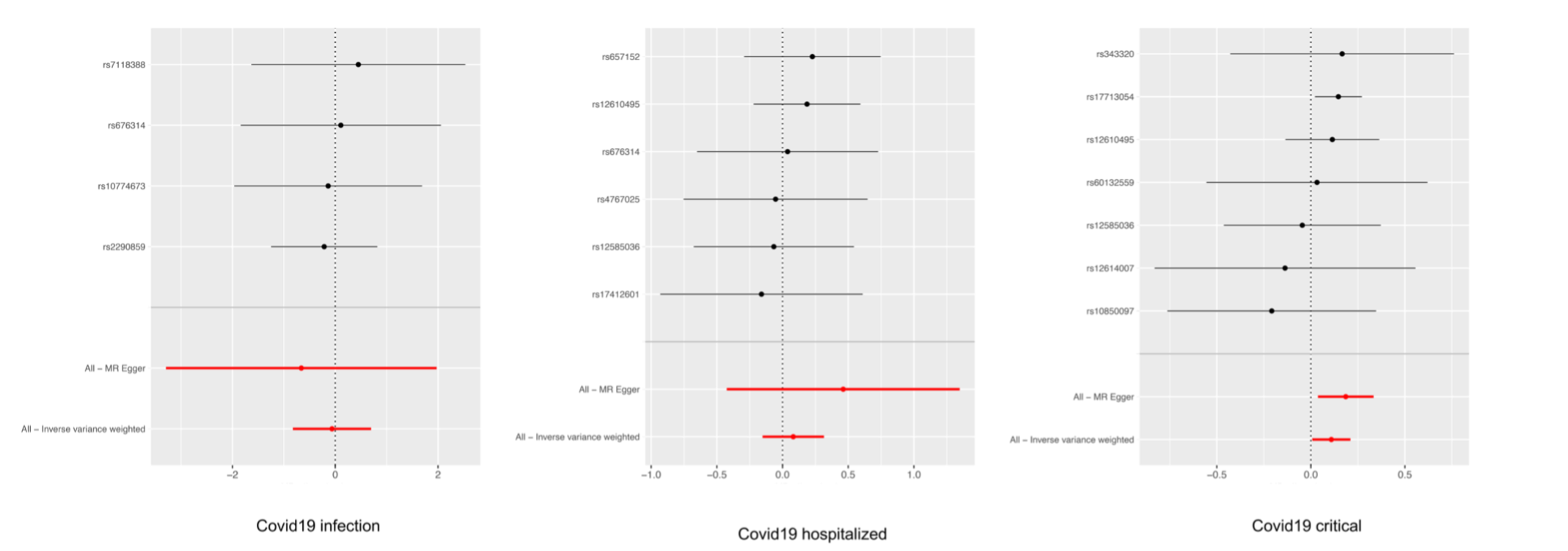


﻿The x‐axis shows MR effect size for COVID‐19 on celiac disease. The y‐axis shows the analysis for each of SNPs.

Abbreviation: COVID‐19, coronavirus disease 2019; MR, Mendelian randomization; SNPs, single nucleotide polymorphisms;
