# Supplementary material for: Critical COVID-19, Victivallaceae abundance, and celiac disease: A mediation Mendelian randomization study
Source: PLoS One. 2024 May 3;19(5):e0301998. doi: 10.1371/journal.pone.0301998 (PMC11068179; doi:10.1371/journal.pone.0301998)
Supplement: Attachment — Submitted filename: Response to Reviewers.docx [file pone.0301998.s003.docx]

Subject: Response to Review and Revisions for Manuscript ID [PONE-D-23-36003R1]

Dear Editor Yasin Sahin and Reviewers,

I hope this message finds you well. I sincerely appreciate the time and effort invested in reviewing our manuscript titled "Critical COVID-19, *Victivallaceae* Abundance, and Celiac disease: A Mediation Mendelian Randomization Study". Your thoughtful insights have been invaluable, and we have carefully considered each point raised during the evaluation process.

**Reply to Review 1**

To address the concerns raised by Reviewer 1 regarding English editing and the focus of the article, we acknowledge the importance of enhancing the clarity and coherence of our manuscript and the focus of gut dysbiosis.

**1. English editing is highly recommended.**

Response: We thank the reviewer for raising the valuable suggestion. Regarding the recommendation for English editing, we want to express our sincere commitment to ensuring the highest standard of language in our manuscript. Recognizing the importance of this aspect, we have sought professional assistance from the company, Editage. In the attached document, you will find certificate of the editing performed on the manuscript.

**2. Focusing the article on the main issues of COVID-19 and celiac disease, and then a hypothesis of gut dysbiosis and Victivallaceae abundance as risk factors**

Response: We thank the reviewer for raising the valuable suggestions. We highly agree with the suggestion to emphasize gut dysbiosis and *Victivallaceae* Abundance as a potential risk factor in the development of celiac disease in critical COVID-19 patients. This perspective has been incorporated into our revised manuscript. We implemented a major revision, reevaluating the general title, abstract, correlative data presentation, and the deep discussion with previous relevant studies.

The abstract and conclusion explicitly establish the connection between COVID-19 and celiac disease for the readers. COVID-19 enhances susceptibility to celiac disease through a reduction in the abundance of *Victivallaceae* abundance. **Detailed information can be found in Bold in Revised Manuscript with Track, specifically line 27 to 32 and 289 to 297.**

In the introduction, we emphasized that genetic factors and a gluten-rich diet are not the only factors in the development of celiac disease. Previous studies have emphasized the link between gut dysbiosis and the development of celiac disease[1, 2]. Understanding the potential influence of COVID-19 on the development or exacerbation of celiac disease, particularly through mechanisms involving gut dysbiosis, has significant implications. **Detailed information can be found in Bold in Revised Manuscript with Track, specifically line 47 to 55.**

Our study suggests that the reduction in *Victivallaceae* abundance could potentially act as a mediator in the relationship between critical COVID-19 and celiac disease. The decrease in *Victivallaceae* abundance might disrupt the delicate balance of immune regulation, creating an environment conducive to autoimmune responses. We further discuss, the potential mechanisms between SARS-CoV-2, gut dysbiosis and celiac disease. SARS-CoV-2 compromises the integrity of the intestinal barrier[3-6]. Reduced populations of specific intestinal bacteria in COVID-19 patients correlate with elevated levels of inflammatory factors [7, 8]. This suggests that gut dysbiosis may contribute significantly to the development of intense and widespread inflammation. Subsequently, inflammatory factors damage intestinal epithelial cells, leading to impairment of the intestinal barrier. The compromised integrity of the intestinal barrier heightens the probability of immune cells being exposed to gluten peptides. This exposure may initiate the autoimmune response characteristic of celiac pathogenesis. Furthermore, in individuals susceptible to celiac disease, gliadin can induce the excessive growth of pathogenic intestinal bacteria, leading to gut dysbiosis and forming a vicious cycle[9]. **Detailed information can be found in Bold in Revised Manuscript with Track, specifically line 243 to 261.**

**Reply to Review 2**

Regarding the points raised by Reviewer 2, we appreciate the guidance on specific sentences and the emphasis on key gut microbiome changes associated with critical COVID infection.

**1. No need to keep the sentences from 231 to 236. It is not adding any strength to the discussion or manuscript.**

Response: We thank the reviewer for raising the valuable suggestion. In the revised version, we have carefully reviewed and omitted those unnecessary sentences.

**2. Literature is not clear that severity of COVID is closely related to no post-COVID syndrome. Even the asymptomatic or mildly symptomatic patients can develop long COVID symptoms. Instead of mentioning post-COVID syndrome, authors might focus more on the critical COVID infection related gut microbiome changes and its potential effect.**

Response: We thank the reviewer for raising the valuable suggestions. Regarding the observation about the clarity in the literature regarding the relationship between COVID-19 severity and post-COVID syndrome, we appreciate the feedback. We have redirected our focus to explore gut microbiome changes with gut microbiome changes and their potential implications, instead of an emphasis on long COVID symptoms.

In the introduction, we found that previous studies have emphasized the link between gut microbiome changes altered (gut dysbiosis) and the development of celiac disease. Understanding the potential influence of COVID-19 on the development or exacerbation of celiac disease, particularly through mechanisms involving gut dysbiosis, has significant implications. **Detailed information can be found in Bold in Revised Manuscript with Track, specifically line 47 to 55.**

In the Discussion section, we discuss in depth that a reduction in *Victivallaceae* abundance may disrupt the delicate balance of immune regulation, creating an environment conducive to autoimmune responses. In addition, we further discuss the potential mechanisms between SARS-CoV-2, intestinal dysbiosis, the gastrointestinal barrier, and celiac disease. SARS-CoV-2 compromises the integrity of the intestinal barrier[3-6]. Previous studies have further demonstrated that gut dysbiosis is present in patients with COVID-19 and may contribute significantly to the development of intense and widespread inflammation[7, 8]. Subsequently, inflammatory factors damage the intestinal epithelial cells, leading to a compromised intestinal barrier. With the integrity of the intestinal barrier compromised, immune cells are more likely to be exposed to gluten peptides. This exposure may trigger an autoimmune response characteristic of celiac disease pathogenesis. In addition, in individuals susceptible to celiac disease, gliadin induce overgrowth of intestinal pathogenic bacteria, leading to intestinal dysbiosis and a vicious cycle[9]. **Detailed information can be found in Bold in Revised Manuscript with Track, specifically line 243 to 261.**

**3. add a small discussion about the role of the presence of genes (particular HLA) to develop celiac disease**

Response: We thank the reviewer for raising the valuable suggestions. We have incorporated a discussion on the role of genes, especially HLA, in the pathogenesis of celiac disease.

Human leukocyte antigen (HLA) genes, particularly HLA-DQ2.5, HLA-DQ 2.2 and HLA-DQ8, are recognized genetic risk factors for celiac disease[10, 11]. Antigen-presenting cells in celiac disease patients express HLA-DQ molecules associated with the disease[12]. Deamination of glutathione produces a negative charge that binds to the disease-associated HLA molecules to form stable peptide-HLA complexes[13-15]. The peptide-HLA complex triggers the activation of gluten-specific CD4+ T cells, leading to the production of pro-inflammatory cytokines[16]. In addition, activated CD4+ T cells interact with B cells to induce antibody production, ultimately leading to destruction of the intestinal villi. In addition, a variety of non-HLA risk loci, many of which are associated with T- and B-cells function, have been implicated in celiac disease[17, 18]. Viral infections and HLA gene variants have a strong interaction[19-21]. Critical COVID-19 may exacerbate genetic susceptibility to celiac disease by affecting HLA-DQ molecules. **Detailed information can be found in Bold in Revised Manuscript with Track, specifically line 215 to 226.**

**4.Can we claim critical COVID infection may trigger/exacerbate the symptoms of celiac disease instead? Given the fact that most celiac disease patients go silent for years until they develop some supporting symptoms, this would worth discussing!**

Response: We thank the reviewer for raising the thoughtful suggestions. The insightful comments have significantly contributed to enhancing the quality and rigor of our research. In this study, we employed the Mendelian randomization approach to investigate associations with disease at the genetic susceptibility level. Claiming the exacerbation of symptoms necessitates additional empirical validation through dedicated experiments. We have discussed the potential mechanisms that may trigger or exacerbate symptoms of celiac disease in critical COVID infections.

During viral infection, the immune system generates a response against specific viral epitopes. Some of these viral epitopes may share similarities with self-antigens in the host, known as molecular mimicry[22]. Cross-reactivity occurs when immune cells, initially activated against viral epitopes, recognize and respond to similar epitopes on host tissues, leading to immune system confusion and autoimmunity[23]. Furthermore, viral infections can cause tissue damage and cell death, resulting in the release of self-antigens not initially targeted by the immune response. The exposure of new self-antigens to the immune system can trigger the expansion of the immune response to encompass these additional epitopes, contributing to epitope spreading. Critical COVID-19 may enhance this molecular mimicry and epitope spreading, potentially triggering celiac symptoms in predisposed individuals. **Detailed information can be found in Bold in Revised Manuscript with Track, specifically line 227 to 238.**

**Reply to Review 3**

For the concerns expressed by Reviewer 3 regarding the association with the *Victivallaceae* family and the reported case numbers, we genuinely acknowledge the merit of the concerns.

**1. It be premature to think that only *Victivallaceae* is associated with Celiac disease.**

Response: We thank the reviewer for raising the thoughtful suggestion. We appreciate the meticulous consideration given to this aspect. Our investigation reveals a significant association between celiac disease and *Victivallaceae* abundance, based on the gut microbial data (211 taxa) provided by Kurilshikov A et al[24]. Indeed, the gut microbiota comprises not only the mentioned 211 taxa but also various others. As correctly pointed out by the reviewer, attributing celiac disease to a single gut microbiota is premature. Existing literature indicates an association between celiac disease and a diverse range of gut microbiota[1, 2], without excluding the possibility of a connection with others gut microbiota. We took great care to acknowledge this limitation within the manuscript. This perspective is thoroughly discussed in our revised manuscript. **Detailed information can be found in Bold in Revised Manuscript with Track, specifically line 272 to 280.** The primary contribution of our study lies in establishing, for the first time, a causal relationship between *Victivallaceae* and celiac disease. This finding serves to reinforce the theory that gut microbiota plays a role in promoting the development of celiac disease.

**2. Unusually high no. of cases as compared to controls.**

Response: We thank the reviewer for raising the thoughtful suggestion. I would like to clarify that the error in the reported number of cases is indeed due to a mistake in our writing process. We recognize the importance of accurate reporting and sincerely apologize for any confusion caused by this oversight. Rest assured, this error has been rectified in the revised manuscript, and we provide the correct and precise information.

The COVID-19 Host Genetics Initiative (<https://www.covid19hg.org/> , Release 7, European ancestry cohorts) provided GWAS summary statistics for severe respiratory symptom (A2: critically ill COVID vs. population), hospitalization (B2: hospitalized COVID vs. population) and COVID-19 infection (C2: COVID vs. population). The GWAS dataset comprised 13,769 cases vs. 1,072,442 controls (A2), 32,519 cases vs. 2,062,805 controls (B2), and 122,616 cases vs. 2,475,240 controls (C2). Summary statistics for gut microbial taxa were obtained from an extensive GWAS analysis involving 18,340 participants with diverse ancestries and ages across 24 cohorts. The available at the [www.mibiogen.org](http://www.mibiogen.org). Summary statistics for Celiac disease were obtained from most comprehensive GWAS to date, analyzing 523,399 SNPs within a cohort comprising 4,533 celiac disease patients and 10,750 healthy individuals. Access to a summary of this dataset is freely available at <https://gwas.mrcieu.ac.uk/datasets/ieu-a-276> . **Detailed information can be found in Bold in Revised Manuscript with Track, specifically line 88 to 94 and 104 to 105.**

We genuinely appreciate the constructive feedback provided by each reviewer, and we are committed to undertaking the necessary revisions to improve the quality and impact of our manuscript. Your guidance is instrumental in shaping the contribution of our research to the field.

Thank you once again for your time and consideration. We look forward to submitting the revised manuscript for your further evaluation.

Best regards,

PhD, Huaqi Guo

Department of Respiratory and Critical Care Medicine, Shanghai Key Laboratory of Tissue Engineering, Shanghai Ninth People's Hospital, Shanghai Jiao Tong University School of Medicine

Email: [guohuaqi814102@163.com](mailto:guohuaqi814102@163.com)

1.	Zoghi S, Abbasi A, Heravi FS, Somi MH, Nikniaz Z, Moaddab SY, et al. The gut microbiota and celiac disease: Pathophysiology, current perspective and new therapeutic approaches. Crit Rev Food Sci Nutr. 2022:1-21. Epub 20220926. doi: 10.1080/10408398.2022.2121262. PubMed PMID: 36154539.

2.	Akobeng AK, Singh P, Kumar M, Al Khodor S. Role of the gut microbiota in the pathogenesis of coeliac disease and potential therapeutic implications. Eur J Nutr. 2020;59(8):3369-90. Epub 20200710. doi: 10.1007/s00394-020-02324-y. PubMed PMID: 32651763; PubMed Central PMCID: PMCPMC7669811.

3.	Eleftheriotis G, Tsounis EP, Aggeletopoulou I, Dousdampanis P, Triantos C, Mouzaki A, et al. Alterations in gut immunological barrier in SARS-CoV-2 infection and their prognostic potential. Front Immunol. 2023;14:1129190. Epub 20230315. doi: 10.3389/fimmu.2023.1129190. PubMed PMID: 37006316; PubMed Central PMCID: PMCPMC10050566.

4.	Jiao L, Li H, Xu J, Yang M, Ma C, Li J, et al. The Gastrointestinal Tract Is an Alternative Route for SARS-CoV-2 Infection in a Nonhuman Primate Model. Gastroenterology. 2021;160(5):1647-61. Epub 20201209. doi: 10.1053/j.gastro.2020.12.001. PubMed PMID: 33307034; PubMed Central PMCID: PMCPMC7725054.

5.	Kariyawasam JC, Jayarajah U, Riza R, Abeysuriya V, Seneviratne SL. Gastrointestinal manifestations in COVID-19. Trans R Soc Trop Med Hyg. 2021;115(12):1362-88. doi: 10.1093/trstmh/trab042. PubMed PMID: 33728439; PubMed Central PMCID: PMCPMC7989191.

6.	Cardinale V, Capurso G, Ianiro G, Gasbarrini A, Arcidiacono PG, Alvaro D. Intestinal permeability changes with bacterial translocation as key events modulating systemic host immune response to SARS-CoV-2: A working hypothesis. Dig Liver Dis. 2020;52(12):1383-9. Epub 20200916. doi: 10.1016/j.dld.2020.09.009. PubMed PMID: 33023827; PubMed Central PMCID: PMCPMC7494274.

7.	Yeoh YK, Zuo T, Lui GC, Zhang F, Liu Q, Li AY, et al. Gut microbiota composition reflects disease severity and dysfunctional immune responses in patients with COVID-19. Gut. 2021;70(4):698-706. Epub 20210111. doi: 10.1136/gutjnl-2020-323020. PubMed PMID: 33431578; PubMed Central PMCID: PMCPMC7804842.

8.	Vabret N, Britton GJ, Gruber C, Hegde S, Kim J, Kuksin M, et al. Immunology of COVID-19: Current State of the Science. Immunity. 2020;52(6):910-41. Epub 20200506. doi: 10.1016/j.immuni.2020.05.002. PubMed PMID: 32505227; PubMed Central PMCID: PMCPMC7200337.

9.	Bernardo D, Garrote JA, Nadal I, Leon AJ, Calvo C, Fernandez-Salazar L, et al. Is it true that coeliacs do not digest gliadin? Degradation pattern of gliadin in coeliac disease small intestinal mucosa. Gut. 2009;58(6):886-7. doi: 10.1136/gut.2008.167296. PubMed PMID: 19433604.

10.	Brown NK, Guandalini S, Semrad C, Kupfer SS. A Clinician's Guide to Celiac Disease HLA Genetics. Am J Gastroenterol. 2019;114(10):1587-92. doi: 10.14309/ajg.0000000000000310. PubMed PMID: 31274511.

11.	Sollid LM. The roles of MHC class II genes and post-translational modification in celiac disease. Immunogenetics. 2017;69(8-9):605-16. Epub 20170710. doi: 10.1007/s00251-017-0985-7. PubMed PMID: 28695286.

12.	Jabri B, Sollid LM. Tissue-mediated control of immunopathology in coeliac disease. Nat Rev Immunol. 2009;9(12):858-70. doi: 10.1038/nri2670. PubMed PMID: 19935805.

13.	Xia J, Sollid LM, Khosla C. Equilibrium and kinetic analysis of the unusual binding behavior of a highly immunogenic gluten peptide to HLA-DQ2. Biochemistry. 2005;44(11):4442-9. doi: 10.1021/bi047747c. PubMed PMID: 15766274.

14.	Henderson KN, Tye-Din JA, Reid HH, Chen Z, Borg NA, Beissbarth T, et al. A structural and immunological basis for the role of human leukocyte antigen DQ8 in celiac disease. Immunity. 2007;27(1):23-34. Epub 20070712. doi: 10.1016/j.immuni.2007.05.015. PubMed PMID: 17629515.

15.	Kim CY, Quarsten H, Bergseng E, Khosla C, Sollid LM. Structural basis for HLA-DQ2-mediated presentation of gluten epitopes in celiac disease. Proc Natl Acad Sci U S A. 2004;101(12):4175-9. Epub 20040312. doi: 10.1073/pnas.0306885101. PubMed PMID: 15020763; PubMed Central PMCID: PMCPMC384714.

16.	Sollid LM, Tye-Din JA, Qiao SW, Anderson RP, Gianfrani C, Koning F. Update 2020: nomenclature and listing of celiac disease-relevant gluten epitopes recognized by CD4(+) T cells. Immunogenetics. 2020;72(1-2):85-8. Epub 20191118. doi: 10.1007/s00251-019-01141-w. PubMed PMID: 31735991.

17.	Dubois PC, Trynka G, Franke L, Hunt KA, Romanos J, Curtotti A, et al. Multiple common variants for celiac disease influencing immune gene expression. Nat Genet. 2010;42(4):295-302. Epub 20100228. doi: 10.1038/ng.543. PubMed PMID: 20190752; PubMed Central PMCID: PMCPMC2847618.

18.	Withoff S, Li Y, Jonkers I, Wijmenga C. Understanding Celiac Disease by Genomics. Trends Genet. 2016;32(5):295-308. Epub 20160310. doi: 10.1016/j.tig.2016.02.003. PubMed PMID: 26972670.

19.	Augusto DG, Murdolo LD, Chatzileontiadou DSM, Sabatino JJ, Jr., Yusufali T, Peyser ND, et al. A common allele of HLA is associated with asymptomatic SARS-CoV-2 infection. Nature. 2023;620(7972):128-36. Epub 20230719. doi: 10.1038/s41586-023-06331-x. PubMed PMID: 37468623; PubMed Central PMCID: PMCPMC10396966.

20.	Ng MH, Lau KM, Li L, Cheng SH, Chan WY, Hui PK, et al. Association of human-leukocyte-antigen class I (B*0703) and class II (DRB1*0301) genotypes with susceptibility and resistance to the development of severe acute respiratory syndrome. J Infect Dis. 2004;190(3):515-8. Epub 20040707. doi: 10.1086/421523. PubMed PMID: 15243926; PubMed Central PMCID: PMCPMC7109646.

21.	Martin MP, Carrington M. Immunogenetics of HIV disease. Immunol Rev. 2013;254(1):245-64. doi: 10.1111/imr.12071. PubMed PMID: 23772624; PubMed Central PMCID: PMCPMC3703621.

22.	Fujinami RS, von Herrath MG, Christen U, Whitton JL. Molecular mimicry, bystander activation, or viral persistence: infections and autoimmune disease. Clin Microbiol Rev. 2006;19(1):80-94. doi: 10.1128/CMR.19.1.80-94.2006. PubMed PMID: 16418524; PubMed Central PMCID: PMCPMC1360274.

23.	Amjadi MF, Parker MH, Adyniec RR, Zheng Z, Robbins AM, Bashar SJ, et al. Novel and unique rheumatoid factors cross-react with viral epitopes in COVID-19. J Autoimmun. 2023;142:103132. Epub 20231111. doi: 10.1016/j.jaut.2023.103132. PubMed PMID: 37956528.

24.	Kurilshikov A, Medina-Gomez C, Bacigalupe R, Radjabzadeh D, Wang J, Demirkan A, et al. Large-scale association analyses identify host factors influencing human gut microbiome composition. Nat Genet. 2021;53(2):156-65. Epub 20210118. doi: 10.1038/s41588-020-00763-1. PubMed PMID: 33462485; PubMed Central PMCID: PMCPMC8515199.
