# Supplementary material for: Critical COVID-19, Victivallaceae abundance, and celiac disease: A mediation Mendelian randomization study
Source: PLoS One. 2024 May 3;19(5):e0301998. doi: 10.1371/journal.pone.0301998 (PMC11068179; doi:10.1371/journal.pone.0301998)
Supplement: Attachment — Submitted filename: Response to Reviewers.docx [file pone.0301998.s004.docx]

Subject: Response to Editors and Reviewers for Manuscript ID [PONE-D-23-36003R1]

Dear Editors and Reviewers,

I hope this message finds you well. I wish to extend my heartfelt thanks for the time and effort you have dedicated to reviewing our manuscript, titled "Critical COVID-19, *Victivallaceae* Abundance, and Celiac disease: A Mediation Mendelian Randomization Study", and for providing constructive feedback. Your thoughtful insights have been valuable, and we deeply value the insights and comments you have shared.

We have thoroughly reviewed each comment and suggestion, and have taken great care to address each point in the revised manuscript. Below, I have outlined our responses to your feedback, detailing the changes we have made.

**Reply to Editors**

**1.The reference about the HLA genes are not associated, please omit that and use the relevant and correct study.**

Response: We are grateful to the editor for their constructive suggestion. We highly agree with the recommendation to modify our references. Accordingly, we have made a revision：Human leukocyte antigen (HLA) genes, particularly HLA-DQ2.5, HLA-DQ 2.2 and HLA-DQ8, are recognized genetic risk factors for celiac disease[39,40]. **Detailed information can be found in Bold in Revised Manuscript with Track, specifically line 217 to 218.**

**2. Changes in references.**

Response: We sincerely appreciate the time and effort editors have dedicated to reviewing my manuscript. Upon further thoroughly review of our manuscript, we found it necessary to update our references.

**(1)** We added the study by Samasca et al. (2021) (PMID：34485888), which better reflects the high prevalence of celiac disease in COVID-19. **Detailed information can be found in Bold in Revised Manuscript with Track, specifically line 43.**

**(2)** We are moved reference that was no relevant to the advantages of MR randomization. **Detailed information can be found in Bold in Revised Manuscript with Track, specifically line 58 and 62.**

**(3)** In the references we have cited, the papers by Christophersen et al. (2022) (PMID：35119226) and Risnes et al. (2021) (PMID：33654213) are related to the immune system in celiac disease rather than COVID-19. Therefore, we have incorporated these references to support the assertion that "Abnormal immune responses play a central role in the celiac disease triggered by gluten ingestion, contributing to inflammation and intestinal damage", and have eliminated any incorrect citations accordingly. **Detailed information can be found in Bold in Revised Manuscript with Track, specifically line 209 to 210.**

**(4)** We added the study by De et al. (2017) (PMID：28913337) to further support the CD4^+^ T-cells argument. **Detailed information can be found in Bold in Revised Manuscript with Track, specifically line 222.**

**(5)** We have rewritten the conclusion section to more clearly articulate the methods and findings of this study, along with the significance of the research, and have removed irrelevant references. **Detailed information can be found in Bold in Revised Manuscript with Track, specifically line 286 to 293.**

**3. Modifications in some part of the manuscript:**

Response: We sincerely appreciate the time and effort editors have dedicated to reviewing my manuscript. After a thorough review of our manuscript, we have implemented several modifications aimed at enhancing the clarity, accuracy, and significance of our study.

**(1)** In accordance with the journal's guidelines, the *corresponding authors should be listed in a specific order: starting with the primary corresponding author, followed by the second, and then the third. Consequently, we have realigned the order of the corresponding authors in the *Corresponding Author to reflect the order indicated by the title's listing of corresponding authors. We would like to clarify that this realignment has been made solely within the *Corresponding Author. The order of the authors, including the corresponding authors, remains consistent with our original submission. **Detailed information can be found in Bold in Revised Manuscript with Track, specifically line 12 to 14.**

**(2)** Based on Mendelian randomization literature[1], we more accurately described "the independence assumption" as: genetic variants should be independent of any confounders that may affect the relationship between the exposure and outcome. **Detailed information can be found in Bold in Revised Manuscript with Track, specifically line 67 to 68.** Additionally, "the exclusion restriction assumption" should emphasize that SNPs affect the outcome only through the exposure pathway. Hence, we revised "SNPs solely influence the outcome through the exposure pathway" to "SNPs influence the outcome solely through the exposure pathway" for clearer articulation of this assumption's meaning. **Detailed information can be found in Bold in Revised Manuscript with Track, specifically line 69.**

**(3)** The F-statistics should be in the range of 53.07255 to 183.849(listed in S2 Table in S2 File). Accordingly, line 153 in the manuscript should be: "*F*-statistics ranging from 53.07 to 183.85". **Detailed information can be found in Bold in Revised Manuscript with Track, specifically line 153.**

**(4)** Exposures and outcomes are considered causally related at a significance level of *P* < 0.05. It is the IVW (*P* = 0.035) and weighted median (*P* = 0.021) methods that could conclude that critical COVID-19 is associated with a higher risk of celiac disease, as demonstrated in Figure 2. **Detailed information can be found in Bold in Revised Manuscript with Track, specifically line 158 to 160.**

**(5)** The results of the Leave-one-out analysis demonstrated the causal relationships of individual SNPs in COVID-19 infection, COVID-19 hospitalize, and critical COVID-19 with celiac disease (S2 Fig in S1 File). According to the conclusions drawn from the article, there is no causal relationship between COVID-19 infection or hospitalized COVID-19 and celiac disease. Therefore, "Leave-one-out analysis showed the effect of individual SNPs on overall causality" could more accurately describes the results of the Leave-one-out analysis. **Detailed information can be found in Bold in Revised Manuscript with Track, specifically line 171.**

**(6)** Upon reviewing our submitted manuscript and reconfirming all the included analysis and data, we realized that there is a minor typographical error in Table 1 of the manuscript. Specifically, the error lies in the *P* value using the weighted mode method when analyzing the causal effect between *Victivallaceae* abundance and celiac disease. This was an inadvertent typographical mistake and we apologize for any confusion it may have caused. I would like to assure you that this error does not alter the findings or conclusions drawn from our study. All analyses and results presented are based on the correct data, and the integrity of our research remains intact. We have rectified at Table 1. **Detailed information can be found in Bold in Revised Manuscript with Track, specifically Table 1 and line 185.**

**(7)** We have rewritten the conclusion section to more clearly elucidate the methods and results of this study, while also emphasizing the significant implication of the research. **Detailed information can be found in Bold in Revised Manuscript with Track, specifically line 286 to 293.**

**Reply to Review 1**

Response: We would like to express my deepest gratitude for the valuable time and effort you have dedicated to reviewing our manuscript.

**Reply to Review 2**

**1. The paragraph starting in the line of 237 should follow the sentence of 240.**

Response: We would like to express my deepest gratitude for the valuable time and effort you have dedicated to reviewing our manuscript. Your constructive suggestion has been valuable to enhancing the quality of our work.

In the revised version, we have positioned the paragraph starting from line 237 to follow the sentence on line 240 in the manuscript. Your expertise has significantly contributed to the improvement of our manuscript.

This study employs the MR approach to avoid uncertainties related to causality. HLA genes are a crucial but not exclusive factor in the development of celiac disease. Earlier investigations have demonstrated an association between celiac disease and gut dysbiosis. Nevertheless, discerning whether gut dysbiosis results from celiac disease or contributes to its development poses a significant challenge.

**Detailed information can be found in Bold in Revised Manuscript with Track, specifically line 238 to 241.**

We genuinely appreciate the constructive feedback provided by editors and reviewers. We have improved our manuscript according to your valuable suggestions. These modifications have significantly enhanced the quality and clarity of our work.

Thank you once again for your time and consideration. We look forward to your further evaluation and hope that our revised manuscript meets the esteemed standards of PLOS ONE.

Best regards,

PhD, Huaqi Guo

Department of Respiratory and Critical Care Medicine, Shanghai Key Laboratory of Tissue Engineering, Shanghai Ninth People's Hospital, Shanghai Jiao Tong University School of Medicine

Email: [guohuaqi814102@163.com](mailto:guohuaqi814102@163.com)

1.	Sekula P, Del Greco MF, Pattaro C, Kottgen A. Mendelian Randomization as an Approach to Assess Causality Using Observational Data. J Am Soc Nephrol. 2016;27(11):3253-65. Epub 20160802. doi: 10.1681/ASN.2016010098. PubMed PMID: 27486138; PubMed Central PMCID: PMCPMC5084898.
